# Burnout among nurses working in social welfare centers for the disabled

**DOI:** 10.1186/s12912-017-0209-3

**Published:** 2017-03-23

**Authors:** Eleni Lahana, Konstantina Papadopoulou, Olga Roumeliotou, Andreas Tsounis, Pavlos Sarafis, Dimitris Niakas

**Affiliations:** 1 Technological Educational Institute of Larissa, Faculty of Nursing, Larissa, Greece; 2 Hellenic Open University, Faculty of Social Sciences, Patra, Greece; 3 Centers for the Prevention of Addictions and Promoting Psychosocial Health of Municipality of Thessaloniki, Thessaloniki, Greece; 4 Department of Nursing, School of Health Sciences, Cyprus University of Technology, 15, Vragadinou Str., Limassol, 3041 Cyprus

**Keywords:** Annexes disabled, Burnout, MBI, Nurses

## Abstract

**Background:**

In the healthcare sector, we often come across the burnout syndrome. It is an occupational syndrome which causes, physical and emotional exhaustion. More information is needed on the dangers of burnout and how often it occurs in healthcare. The purpose of this study was to investigate burnout and factors associated with the syndrome among nurses working with people that are mentally challenged.

**Methods:**

A cross-sectional survey was conducted, among 180 nurses working in public health centers for the disabled in multiple regions of Greece. A self-administered questionnaire with questions about socio-demographic and work-related characteristics was used, as well as the Maslach Burnout Inventory (MBI) for burnout assessment. Univariate and multivariate analyzes were performed.

**Results:**

The burnout dimensions of emotional exhaustion (Mean = 31.36) and depersonalization (Mean = 11.27) were at high levels while personal accomplishment was at low levels (Mean = 44.02). Female nurses had a higher personal accomplishment score (Mean = 44.82, *p =* 0.047) than men (Mean = 42.10, *p =* 0.047). Marital status, daily routine and relationships with supervisors were significantly related with emotional exhaustion and personal accomplishment and professional experience with higher levels of emotional exhaustion and depersonalization. Reason for professional selection was an independent predictor for depersonalization and personal accomplishment, with those that have selected the nursing profession randomly or because of the fear of unemployment having higher scores. Moderate relationships with colleagues was an independent predictor for all burnout dimensions.

**Conclusions:**

Nurses working in services for people with intellectual disabilities in Greece show increased burnout levels. Burnout can be prevented by offering more opportunities for professional advancement and education, new ways to provide supervisor support, provide incentives for nurses to initiate or participate in innovative programs. Specific training on conflict resolution, collaboration, reinforcement and stress coping techniques must be implemented.

## Background

The burnout syndrome is an occupational illness relatively common among health professionals. Empirical studies have demonstrated that burnout has many adverse effects on the physical and emotional health of health care professionals, such as physical fatigue, cardiovascular disorders and other organic diseases like anxiety, depression, and loss of motivation [1–6]. It is a syndrome that may potentially affect an organization’s infrastructures, leading to decreased productivity in the workplace and to the deterioration of the quality of healthcare provided, which can have a negative impact on the health system in general [1, 2, 7].

The term of burnout was introduced by Freudenberger in 1974, defining a condition of physical and mental energy depletion, as a response to ongoing exposure to occupational stress factors. It is a multidimensional construct that involves a number of parameters such as emotional exhaustion, depersonalization, and lack of perceived personal accomplishment [2]. In particular, Emotional Exhaustion (EE), burnouts’ main stress dimension, refers to feelings of being emotionally overextended and it mainly derives from work overload and personal conflict in the work environment. DePersonalization (DP) on the other hand, represents the interpersonal dimension of the syndrome and refers to being negative and detached when communicating with other people. Whereas, lack of perceived Personal Accomplishment (PA), refers to a sense of low professional achievement and productivity, and represents the self-evaluation aspect of burnout [2].

Efficiently identifying and determining the risk factors and the variables comprising and significantly affecting the burnout phenomenon can facilitate the introduction of effective practices in human resources management and required changes in an individual’s relationship with work, resulting in evidence-based interventions [8].

A number of personal, interpersonal and organizational stressors lead to the development of burnout syndrome. Age, work experience and personality traits like insecurity, cowardice, emotional instability, low stress tolerance, defense mechanisms and self-control, are some of the personal stressors that may positively contribute to increased burnout [9, 10]. On the other hand, the most important and common organizational stressors are lack of staff, work overload, unnecessary bureaucracy, uncertainty for the results of provided healthcare, contact with death, inadequate health and safety services, the type of leadership, bad management, lack of work recognition and sense of loss of control in the working environment [9, 11–14]. More specifically, previous studies have reported that the less experienced nurses, with less education, and no social support are more susceptible to burnout [15, 16]. Other organizational stressors like unsupportive staff communication, shift changes, lack of continuing education, uncertainty about course of treatment used and inability to make independent decision, also play an important role towards the development of burnout syndrome [4, 8, 12, 17, 18].

Regarding its prevalence, nursing personnel is more susceptible to burnout compared to other health-care workers [19]. In a large survey in which 43,000 nurses from more than 700 hospitals in the United States, Canada, the United Kingdom and Germany took part, a large percentage of nurses ranging from 30 to 40% had higher scores related to the norms for medical workers published by the developers of the Maslach Burnout Inventory [20]. However, previous studies report differences in burnout among nurses employed in different sectors and differences in its defined dimensions. The results of a systematic review of 27 studies concerning burnout among oncology nurses show that they present high levels of EE and reduced levels of PA [12]. Furthermore, with regard to a literature review by Embriaco et al. investigating burnout among Intensive Care Unit’s nurses, one third of the critical care nurses presented severe burnout syndrome [21]. While, in a comparative study conducted in medical, psychiatric, surgical and burn wards of a hospital in Iran, results indicated that psychiatry nurses showed higher levels of emotional exhaustion and depersonalization, in comparison to all the others sectors in the study, indicating that burnout may be affected differently by various clinical settings [22].

Consequently, when predicting burnout in health care professionals working with intellectually disabled patients, the challenges confronted by those working in such specialized care units must be taken into account. Patients with intellectual disabilities (ID) are commonly in need of both instrumental and emotional support. In many cases communication is hindered by their verbal and social communication skills [23, 24]. Therefore, workers’ direct care role also includes a variety of other supporting activities, including hygiene and health maintenance, managing finances and help with the use transportation and access to community [25, 26]. In the literature, ID support staff for ID, are commonly confronted with a unique set of stressors such as, challenging behavior and interpersonal demands. These are behaviors that may put at risk the physical safety of the staff and the patients. Decreased access to community facilities and inappropriate behavior as well as work and patient relationships that are more emotionally demanding [27, 28].

Few studies have investigated stressors in association with burnout and those were not fully conclusive on the causal nature of stressors experienced by healthcare professionals when working with the ID and burnout [23]. Some reports suggest that nurses for the intellectually disabled patients, may not be at increased risk of burnout compared to the nursing staff in other areas. [29]. In accordance, the findings of a systematic review that included fifteen studies from six countries, showed that the levels of burnout did not differ to a significant degree from those of the normative sample as described by Mashlach, with the exception of Depersonalization scores that were higher [29]. As far as factors and determinants of burnout of personnel in units for people with disabilities, challenging client behavior is the most frequently reported stressor [23, 30]. In addition, personality traits like neurotic behavior, extraversion and being very self conscious may be predictors of burnout, as is also lack of social support [31]. Finally, organizational factors like role ambiguity, role conflict and inadequate managerial support are significantly associated with burnout levels [25, 31, 32].

According to a study that was conducted in 2000, comparing Southern European countries and their health care services for the intellectually disabled, Greece showed the lowest development in community care in comparison to the other European countries [33]. In particular, the majority of the health care institutions for the intellectually disabled are located in the greater capital area. In most cases, they accept both children and adults, while on numerous occasions the same institution provides health care services for mental, physical, motor and socially handicapped individuals [33]. Despite the de-institutionalization efforts in the past two decades, very few changes have taken affect. The country’s financial crisis in 2010, has lead to healthcare budget reductions, this put new barriers to the much needed interventions of those infrastructures. Only between 2009 and 2013 total health expenditures in Greece dropped by 31.9% [34] impacting negatively on the health care services as a whole. Part of these reductions resulted in the merger of many public welfare health care centers for the disabled, which evidently formed bigger, centralized in the prefectures, health care services for the disabled in general (children and adults with mental, motor and other types of disabilities). This resulted in the loss of their rehabilitation or educational character and limited their services to solemnly supporting the disabled individuals with financial problems, in their daily routines. In addition, these health care centers in many cases, lack some of the health care specialties required, are usually understaffed and the workers are expected to perform numerous professional roles.

This is why, this study attempted to investigate the level of burnout, with a focus on the personal as well as on the organizational determinants associated with the syndrome, among nurses employed in the public health care services for the ID. As far as we know, this is the first study concerning burnout syndrome amongst nurses who care for people with intellectual disabilities in Greece. In addition, similar studies in other countries do not differentiate between nursing staff and other professionals burnout levels.

## Methods

### Participants

The participants of the current study were registered nurses, assistant nurses and carers, working in regional annexes of social welfare centers for the disabled in Greece. All existing government run regional annexes for the disabled, except one, were included in the study and these are under the organizational and financial supervision of the Hellenic Ministry of labor, social security and social solidarity (previously under the supervision of the MoHaW). These specialized health care units for the disabled arose after the integration of previous health care structures for the disabled and in their current form they provide health care services to adults with mental and motor disabilities. In total, 15 centers from southern and western Greece regions participated in the study. Questionnaires were sent by e-mail to the heads of the aforementioned annexes and then to all health workers. The analysis was based on a dataset of 302 participants with a total of 180 questionnaires being fully completed, exhibiting a response rate of 59.60%. The mean age of the responders was 42.07 (SD = 6.71) and the majority of them were women (71.11%).

### Procedure and ethical considerations

The Ethics Committee of the Hellenic Open University and of each one of the units of the disabled granted permission for conducting the research. Anonymous questionnaires were filled in by the participants on their own time. Each questionnaire was accompanied with a brochure explaining the purpose and the anticipated outcomes of participation to the specific research. The employees were informed that completing the questionnaire would be interpreted as informed consent. All data was confidential, only researchers had access. The investigation was carried out in September 2014 and burnout was assessed with the MBI questionnaire. The three MBI scales were the dependent variables of the study. The questions estimating the socio-demographic and occupational variables were assessed as the independent variables.

### Research instruments

#### Socio-demographics and occupational parameters

The first part of the questionnaire contained questions regarding socio-demographic and work-related characteristics of the sample. Socio-demographic characteristics included gender, age, marital status, number of children and educational level. Occupational variables included reasons for working as a nurse, professional experience, income satisfaction, organized work, relationships with colleagues and superiors, staff adequacy, daily routine (a sense of tediousness), the types of shifts and the number of shifts.

#### Burnout

The Greek version of Maslach’s Burnout Inventory (MBI) [MBI-Human Services Survey (MBI-HSS)] was used for measuring burnout levels [32]. MBI is a seven point Likert scale of 22 items (0: never, 6: every day). The 22 questions comprise three grouped scales: a) emotional exhaustion, measuring feelings of being emotionally overextended and exhausted by one’s work, b) depersonalization, measuring an unfeeling and impersonal response towards recipients of one’s service, care treatment or instruction and c) personal accomplishment, measuring feelings of competence and successful achievement in one’s work. Questions 5, 10, 11, 15, 22 refer to emotional exhaustion, 4, 7 9, 12, 17, 18, 19, 21 refer to personal accomplishment and the rest refer to depersonalization. To discriminate between the levels of each dimension of burnout, scores provided by the Greek version of MBI were used, referring to burnout dimensions in health care professionals in Greece, classifying burnout in low, moderate and high category [35]. In detail, for emotional exhaustion, the cut-off point for a high level was ≥31; for depersonalization, it was ≥11; and for personal accomplishment, it was ≤35. Therefore, high scores for emotional exhaustion and depersonalization, and low scores for personal accomplishment were regarded as indicative of burnout. (Table 2). A-Cronbach was 0.83 for this study.

**Table 1:** Socio-demographic and occupational characteristics of the sample

Socio-demographic characteristics	TOTAL Number	(*N =* 180) %
Gender		
Men	52	28.89
Women	128	71.11
Age		
20–40	69	38.33
41–50	95	52.77
51–60+	16	8.88
Marital Status		
Married	131	72.78
Single	27	15.00
Widowed/Divorced/Separated	22	12.21
Children		
None	39	21.66
One child	74	41.11
Two children	27	15.00
Three or more children	40	22.22
Education		
Low	131	72.78
University	46	25.55
Higher postgraduate studies	3	1.66
Occupational variables		
Reason for working as a nurse		
Fear of Unemployment (Easy work placement)	49	27.22
Randomly	48	26.66
Love for the profession	83	46.11
Satisfaction from Income		
No/A bit	154	85.55
Very	22	12.22
A lot	4	2.22
Staff Adequacy		
Yes	10	5.56
No	170	94.44
Organization Satisfaction		
No	46	25.55
A bit	59	32.78
Enough	65	36.11
Much	10	5.55
Relationship with colleagues		
No/A bit	46	25.55
Moderately	103	57.22
Very/Much	31	17.22
Relationship with Superior		
No/A bit	50	27.78
Moderately	101	56.11
Very/Much	29	16.11
Daily routine		
Always	54	30.00
Sometimes	78	43.33
Rarely/Never	48	26.66
Night Shifts per 15 days		
1–2	65	36.11
3–5	59	32.78
> 5	11	6.11
None	45	25.00
Professional Experience		
0–10 years	58	32.22
11–20 years	69	38.33
> 21 years	53	29.44

**Table 2:** Mean scores and percentage of sample as distributed to the Burnout Syndrome scales and comparison of the mean scores and SD according to demographic and occupational characteristics

	Emotional Exhaustion	Depersonalization	Personal Accomplishment
	Mean (SD)	Mean (SD)	Mean (SD)
In total (*n =* 180)	31.36 (11.60)	11.27 (6.05)	44.02 (8.41)
^a^Low	≤20 (13.30%)	≤5 (28.90%)	≥42 (76.70%)
Moderate	21–30 (26.10%)	6–10 (31.10%)	41–36 (21.70%)
High	≥31 (60.60%)	≥11 (40.00%)	≤35 (1.70%)

^a^Low, Moderate and High scores are in accordance to cut-off scores for the Greek population

### Data analysis

Descriptive statistics were conducted for the socio-demographic characteristics (gender, age, marital status etch.) and the occupational parameters (staff adequacy, organizational satisfaction etch.). The Maslach questionnaire was investigated with multifactorial analysis, identification of the three factors that comprise the three grouped dimensions of the MBI [emotional exhaustion (EE), depersonalization (DP) and personal accomplishment) and the Cronbach’s Alpha coefficient estimating internal consistency of the MBI scales.

Univariate analysis followed by multiple regression analysis was employed in an attempt to identify independent risk factors for the three MBI dimensions according to the socio-demographic and occupational characteristics. Variables related to burnout at *p =* 0.10 level were included in the final models [36]. Because all variables but age were categorical, dummy variables (1–0 values) were created for every categorical variable (dummy variables number equal to categories minus one for each categorical variable), in order to enter the linear regression model.

Analyses were performed with the statistical package SPSS 17 (SPSS Inc, Chicago, IL, USA).

## Results

### Socio-demographic and occupational characteristics

The analysis is presented in Table 1. The majority of the respondents were women (71.11%), with a mean age of 42.07 (6.71) (52.77%), married (72.78%) with one child (41.11%). Only 25.55% of the responders were University graduates, while the majority of them (72.78%) were at a lower education level (technological educational institutes) and 48.90% were assistant nurses. Most of the participants (46.11%) chose the profession out of love and a personal need to provide services to humans and almost three quarters of them reported none to low income satisfaction in relation to the provided services (85.55%).

Furthermore, the majority of nurses (94.44%) reported no staff adequacy, and yet 36.11% of them stated satisfaction for the current working conditions. As far as relations with colleagues and superiors, most workers were moderately to very satisfied, with a percentage of 57.22 and 56.11%, respectively, while 17.22 and 16.11% were very satisfied respectively. The 30.0% of the sample report daily routine (always) and 43.33% of the sample believe that sometimes there is daily routine, with a 36.11% having 1–2 night shifts and 6.11% over 5 shifts. Concerning professional experience, 38.33% of the participants have an average work experience of 11–20 years.

### MBI mean scores according to the demographic and occupational characteristics

The mean scores for the MBI scales of the burnout syndrome were estimated and the percentages of the sample according to the mean scores are presented in Table 2. The majority of the responders were positive for burnout syndrome, presenting high scores for the two scales of emotional exhaustion [31.36 (11.60)] and depersonalization [11.27 (6.05)] and low score for personal accomplishments [44.02 (8.41)]. Low, moderate and high burnout levels are defined according to cut-off scores for the Greek population [35].

### Prediction models for the burnout dimensions and association of the dimensions with occupational characteristics

According to the univariate analysis presented in Table 3, satisfaction from the organization and professional experience were significantly related to EE and DP, while reasons for working as a nurse were related to depersonalization and personal accomplishment. Relations with colleagues affected all burnout dimensions. Relations with superiors and daily routine were significantly associated with EE and PA. EE also significantly associated with marital status and income, DP with gender and educational level and PA also with gender and marital status.

**Table 3:** Regression model for burnout dimensions

Demographic and working Characteristics	Emotional Exhaustion		Depersonalization		Personal Accomplishment	
	R^2^	*p*	R^2^	*p*	R^2^	*p*
Gender	0.007	0.281	**0.016**	**0.093**	***0.022***	***0.047***
Age	0.025	0.791	0.015	0.762	<0.001	0.791
Marital Status	**0.030**	**0.086**	0.015	0.432	***0.063***	***0.009***
Children	0.015	0.440	0.003	0.915	0.031	0.132
Higher Education	0.002	0.865	***0.035***	***0.042***	0.012	0.424
Specialty	0.002	0.827	0.025	0.105	0.018	0.193
Reasons for working as a nurse	0.019	0.184	***0.043***	***0.021***	***0.068***	***0.002***
Income satisfaction	**0.032**	**0.056**	0.015	0.256	0.004	0.704
Organization satisfaction	***0.034***	***0.041***	***0.047***	***0.015***	0.016	0.234
Relationship with colleagues	***0.104***	***<0.001***	***0.054***	***0.008***	***0.059***	***0.001***
Relationship with superiors	***0.068***	***0.002***	0.021	0.147	***0.044***	***0.019***
Daily routine	***0.106***	***<0.001***	0.009	0.650	**0.029**	**0.075**
Night shifts	0.027	0.185	0.023	0.250	0.023	0.248
Professional experience	**0.030**	**0.066**	***0.039***	***0.030***	0.014	0.275

-All variables included in the multiple regression models for burnout dimensions are highlighted

-Statistical significant relations are both highlighted and in italics

According to the multivariate analysis of the burnout dimensions (Table 4), income satisfaction, relationship with colleagues and longevity independently related to EE. Low income satisfaction and moderate colleague relations increased EE, while few years of service decreased EE. In the same context, independent predictors of DP were organized work satisfaction, relations with colleagues, longevity and profession selection reasons. Furthermore, low organized work satisfaction and moderate relations with colleagues increased DP, while few years of service decreased DP. Moreover, if the nursing profession was selected randomly or due to unemployment fear, DP increased. PA dependent upon reasons for professional selection and relationship with colleagues i.e. if the nursing profession was selected randomly or due to unemployment fear, or relationship with colleagues was moderate, then PA decreased.

**Table 4:** Multivariate regression model for burnout dimensions

EE model (R2 = 0.185	Emotional Exhaustion									
	Income satisfaction: No/a bit		Moderate relationship with colleagues		Longevity:0-10 yrs					
Intercept^1^	Coefficient B (CI)	*p*	Coefficient B (CI)	*p*	Coefficient B (CI)	*p*				
27.5 (20.9–34.0)	5.3 (0.36–10.3)	0.048	8.1 (3.9–12.9)	<0.001	–4.5[–8.6–(–0.32)]	0.035				
DP model (R2 = 0.193)	Depersonalization									
	organization satisfaction: No/a bit		Moderate relationship with colleagues		Longevity:0–10 yrs		Profession selection reasons: fear of unemployment		Profession selection reasons: randomly	
Intercept	Coefficient B (CI)	*p*	Coefficient B (CI)	*p*	Coefficient B (CI)	*p*	Coefficient B (CI)	*p*	Coefficient B (CI)	*p*
10.5 (6.6–14.4)	2.7 (0.8–4.6)	0.005	3.0 (1.0–5.0)	<0.0001	–2.3 [–4.4–(–1.6)]	0.035	2.5 (0.5–4.4)	0.014	2.2(0.2–9.2)	0.033
PA model (R2 = 0.167)	Personal Accomplishment									
	Profession selection reasons: fear of unemployment		Profession selection reasons: randomly		Moderate relationship with colleagues					
Intercept	Coefficient B (CI)	*p*	Coefficient B (CI)	*p*	Coefficient B (CI)	*p*				
41.6 (35.6–47.6)	–3.9 [(–6.7)–(–1.1]	0.007	–3.4 [(–6.3)–(–0.6),	0.019	–4.2(–7.1)–(–1.35),	0.004				

*EE* Emotional Exhaustion, *DP* Depersonalization, *PA* Personal Accomplishment

^1^Example: If a participant reported no satisfaction with income, moderate relationship with colleagues and 0–10 longevity, then EE = 27.5+ 5.3 × 1 + 8.1 × 1–4.5 × 1 = 27.5 + 5.3 + 8.1-4.5 = 36.4

## Discussion

This study revealed that burnout among nurses working in social welfare annexes for the disabled in Greece is relatively high. In particular EE and DP were high compared to the Greek cut-off scores for Maslach’s Burnout Inventory (MBI) [35], while PA was in lower values. This is not in line with the results of previous studies that were shown to have scores around the mean of the normative samples for the MBI [23, 29], suggesting similar levels of burnout in health care professionals serving intellectually disabled patients and other populations, therefore suggesting a lack of cause and effect between challenging behavior amongst individuals with intellectual disabilities and burnout amongst supporting staff [29]. However, most studies in the field of health care for the intellectually disabled individuals assess burnout collectively across the support staff and fail to individualize burnout levels across the health care professional spectrum (nurses, social scientists, doctors amongst others). Therefore, comparisons can only be made under this limitation.

Gender did not significantly associate with EE and DP, although past research proved that gender (being male) could significantly predict the levels of DP [25] and EE [27], among professionals working with disabled individuals. On the contrary, in the dimension of PA, women showed a higher interest to gain objectives and increase perspectives than men. This is possibly due to the fact that traditionally in Greece the nursing profession is considered a feminine profession [37] and that variables other than work characteristics are more important in accounting for PA levels [38]. It is noteworthy that in our study, male nurses were represented by a relatively low percentage of 28.9% in contrast to women that were the majority (71.1%) of the responders.

Age did not reveal significant associations with any of the three dimensions of burnout syndrome, diverging from the results of previous studies [39, 40]. The development of resilience to everyday stressors of individuals aged 50 years or older could explain the lack of association for age [41]. Also, marital status and level of education were in small part associated with the burnout dimensions. Burnout has been found to be higher among single workers and workers with no children, compared to those nurses that are married and those with children [42]. Implicating, lower burnout in professionally orientated nurses with families, because they perceive work differently.

In addition, professional experience affected the dimensions of DP and EE, which is in agreement to a previous study conducted in the UK [43]. Increasing work experience occasionally increases emotional exhaustion, which in turn may increase the levels of depersonalization and lower the levels of personal accomplishment [44], whereas other studies show no association of burnout with age and years of work experience [27, 28].

Among the personal characteristics, the most important independent predictor for the DP and PA dimensions was the reason for choosing nursing profession. According to the study’s results, when the nursing profession was selected randomly or due to unemployment fear, DP increased, while PA decreased. In today’s Greece’s economy, choosing nursing as a profession randomly, is because career guidance and job counseling are not offered in school. Seventeen year old students make their selection solemnly by completing a computerized bulletin. Being well informed when choosing nursing as a profession may avert burnout, since in most cases it is chosen to satisfy the need to help other people. This may lead to disappointment, the expectations of their professional role and the reality of the job’s demands may be different, which in many instances relates to increased occupational stress [45]. The lack of a definitive professional role and job description in healthcare infrastructures for the mentally challenged, in association with increased workload, may increase the need of the nursing staff to distance themselves because their professional accomplishments do not meet their perceived work objectives.

Additionally, as demonstrated by the multiple regression models, occupational stressors were by far the most important predictors of burnout in our study. However, we need to be cautious, since only 21% of burnout is explained by the study’s prediction models, which in itself raises the need for further in-depth analysis of all possible parameters related to burnout.

In detail, relations were satisfactory with colleagues associated with all three dimensions of burnout syndrome. Quality of relations with supervisors, affected both EE and PA. Quality interpersonal relations among colleagues and between personnel and supervisors, can increase the levels of perceived workplace support, which potentially, significantly, decrease burnout levels [24]. In a study conducted among 80 disability support workers in Australia, it was demonstrated that less workplace support related to greater EE and DP [23]. Moreover, work relations among team members with superiors, between staff nurses, head nurses or managers, play a pivotal stressor role that can increase burnout [46]. Hence, negative interpersonal interactions with management and lack of support from colleagues, positively contributes to increased levels of the burnout phenomenon [47].

Finally, having a daily routine lead to higher levels of EE and lower levels of PA. The existence of a daily routine potentially reduces job interest and may significantly increase emotional exhaustion [48, 49]. In general, routine-based nursing practices may have a negative impact both on the nurses’ sense of self worth and society’s perception of nursing [48].

### Limitations

This study had certain limitations that should be taken into account. Firstly, there was a reduced response rate from some of the centers. In particular, areas of western and eastern Attica, Rethymno in Crete and Larissa had a very low number of respondents, with northern Aegean (Lesvos) showing 0% response rate. Also, there was a time delay to collect all the questionnaires from all the prefectures of Greece and the administrative and transportation costs were not funded, limiting therefore the areas included. Also, in order to measure a true prediction, a longitudinal research design should be applied. Finally, the sample size is relatively low, but this was mainly due to the possible mistrust of the nursing staff about the response confidentiality, regardless of full confidentiality reassurance and this was further limited because the analysis was based on the prerequisite that the participants carefully read the questionnaires, fully understood the questions and replied with honesty, therefore they should be considered with caution.

## Conclusions

To our knowledge, this was the first study on burnout among nurses working with the mentally challenged in Greece and one of the few focusing on emotional exhaustion among nurses in such a specialized field worldwide, since the majority of empirical research measures burnout in healthcare for the disabled as a whole. According to the main findings EE and DP were high, while PA was in lower values compared to Greek cut-off scores for Maslach’s Burnout Inventory. Concerning individual characteristics, female nurses had a higher PA score and scored lower for DP than men. Marital status significantly related to EE and PA and professional experience showed higher levels of EE and DP. The reason of selecting the nursing profession was an independent predictor for DP and PA, with those selecting nursing randomly or because of the fear of unemployment demonstrating higher scores to those that chose the profession out of need to help others. As far as interpersonal relationships and organizational characteristics are concerned, reporting a daily routine and low quality of interrelations with supervisors significantly increased EE and PA, while moderate relations with colleagues was also an independent predictor for all burnout dimensions.

On the basis of the above findings, various practices for burnout prevention could be potentially adopted. Given that this study found that the incentives related with profession selection might lead to increased burnout levels, measures for enhancing professional interest must be taken. Continual education and training sessions may contribute positively to this direction, whereas the reorganization of the nurses’ work may meet the profession’s role expectations and decrease stress. Creating a positive meaning concerning nurses’ role is a major challenge that may lead to reassessment of personal goals and perspectives and consequently reduce burnout levels. This can also benefit those who work many years, who according to our findings also confront greater EE and DP levels.

Secondly, since all burnout dimensions are positively associated with the quality of interpersonal relations especially with colleagues, measures for the improvement of communication and teamwork must be implemented on the health care infrastructures for the mentally challenged. Workshops facilitating the sharing of feelings, the normalization of experience, the strengthening of collaboration, the management of conflict resolution and positive reappraisal can create a healthy working environment [50].

In accordance, improving supervision for efficient nurse support can be another important intervention for the management of organizational burnout. Clinical supervision has well documented benefits for nurses working with the mentally challenged. [51].

Finally, measures at the institutional level, including staff recognition policies, planning more staff breaks, and providing incentives for participation and autonomy may contribute to change the daily routine that is associated with higher EE and lower PA levels, while other studies further support the notion that supportive work environments can positively reduce and even prevent the burnout phenomenon [9, 52, 53].
